# The Lower Orbital Septum Revisited: A 3-Dimensional Structure Determines Aesthetic Presentation and Impacts Operative Rejuvenation

**DOI:** 10.1093/asjof/ojaf050

**Published:** 2025-06-03

**Authors:** Jared M Davis, Joshua Choo, Ronald Brooks, Douglas Gossman

## Abstract

**Background:**

The anatomic basis of lower eyelid bulging remains enigmatic, and the concept of compartmentalization is often used to describe bulge location but does not completely explain the cause of periorbital aging.

**Objectives:**

The authors of this study aim to explore lower septal structure, including the concept of adipose tissue compartmentalization, and assess relevance to aesthetic presentation and operative rejuvenation.

**Methods:**

The inferior orbital septa of 10 cadavers (20 lids) were dissected with magnification, with special attention to contiguous tissues, such as orbital fat, orbicularis oculi muscle, tarsus, and inferior oblique muscle. The cadaveric specimens were 71 to 83 years old (mean = 75). Subsequent comparative observations were made in 63 consecutive patients undergoing lower lid blepharoplasty for eyelid distention. The age range was 35 to 82 years (mean = 63).

**Results:**

Both cadavers and patients demonstrated a loose anterior membrane deep to the postorbicular fascia and discrete, transverse ligamentous elements deep to the anterior membrane. In surgical patients, evaluation of the posterior adipose tissue space disclosed a posterior septal membrane that joined the anterior septal membrane at the superior transverse ligament, creating a discrete compartment isolating the adipose tissue from the eyelid protractors and retractors. Variation in the gross fibrous characteristics of septal constituents was observed in both cadavers and operative patients, and this accounted for observed patterns of clinical presentation.

**Conclusions:**

Lower lid topography is predictive of the fibrous character of the underlying septal components, and topography is useful for surgical planning.

**Level of Evidence: 4 (Therapeutic):**

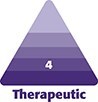

Specific structural characteristics of lower eyelid bulges have long been topics of interest and debate.^1,2^ Among reported causes of bulging in the aging lower eyelid are increased fat volume, dehiscent or herniated orbital septum, and age-related modification of osseous orbital structure.^3-10^ Compartmentalization is a descriptive concept used to locate bulges on the lower eyelid's surface.^11,12^ However, unlike facial adipose tissues, for which true physical compartmentalization is established, specific 3-dimensional (3D) adipose tissue boundaries are as yet undefined.^13^

Clinical and cadaveric studies of lower eyelid septal anatomy reported to date reveal a complex structure and a varied and conflicting descriptive nomenclature. Whitnall's 1945 report characterized the inferior septum of cadavers as a delicate fibrous blanket covering the suborbicular eyelid.^2^ Subsequently, Manson et al described a fibrous septal component which he termed the arcuate expansion. It originated from the lateral arcus marginalis with connections to Lockwood's ligament and the inferior oblique fascia.^14^ Codner and Hanna observed a similar structure dividing lower eyelid adipose tissue into medial and lateral compartments.^15^ Weinberg and Baylis observed a fibrous extension of the inferior oblique muscle and named it the arcuate expansion of the inferior oblique (AEIO).^16^ A fibrous septal structure extending transversely from the medial to the inferolateral orbital rim was also reported by Tanaka, who called it the anterior superior transverse ligament (STL).^17^ Hwang described a fan-like band, deep to the orbital septum and anterior to the inferior oblique muscle, that arose from the inferolateral rim and inserted into the medial canthal tendon.^18,19^

These reports served to advance understanding of the orbital septum, yet additional investigation is needed to fully explain the varied patterns of eyelid distension. Beyond elucidating the basis of aesthetic deformity, septal anatomic knowledge is relevant to operative intervention because its manipulation is a component of contemporary lower blepharoplasty, and understanding the anatomy is pertinent to successful blepharoplasty as well as avoidance and management of select blepharoplasty complications.^20-30^

## METHODS

The lower orbital septum was evaluated first in 10 fresh cadavers (20 lids). Loupes (4.5×) and a surgical microscope–aided elevation of a myocutaneous flap for broad septal exposure and evaluation. Septal components were sequentially identified and photographed.

Subsequently, we studied 64 consecutive lower eyelid blepharoplasty patients (128 lower lids). Being observational in nature, the study did not alter patient treatment and was consistent with the Declaration of Helsinki. A canthofornix exposure, consisting of lower lateral canthal release and deep-fornix conjunctival incision followed by caudal distraction of the conjunctiva/retractor composite, provided a view of the intact septum comparable with that of the cadaver study with added evaluation of the posterior adipose space.^31^ Loupe magnification (4.5×) assisted dissection. Except for skin incisions, monopolar electrocautery with Colorado tip cautery and Penfield 4 elevator were the exclusive dissection instruments. Objectives were assessment of concordance with cadaveric findings and correlation of operative anatomic findings with specific eyelid distention patterns.

## RESULTS

### Cadaver

**Figure 1.**
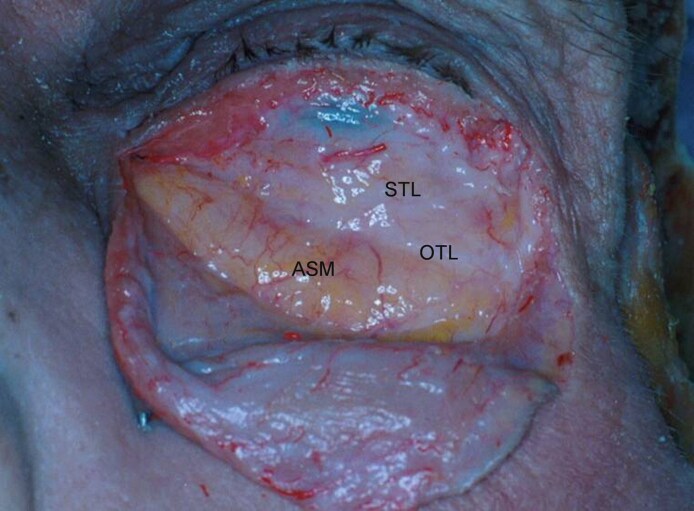
The diaphanous cadaver anterior septal membrane (ASM) allows fat to prolapse over the entire inferior orbital space. Firm superior transverse (STL) and oblique transverse (OTL) ligaments limit its upward translation, whereas firmness of the temporal ASM prevents superotemporal distention. The postorbicular fascia covers the orbicularis oculi muscle.

**Figure 2.**
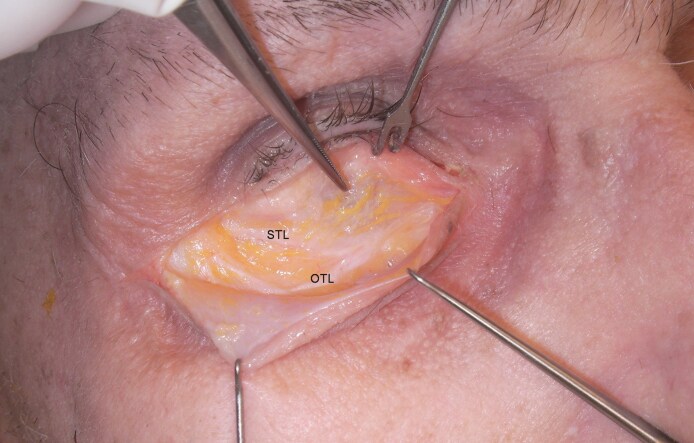
The superior transverse ligament (STL) crosses the eyelid 4.0 to 6.0 mm below the inferior tarsal margin. It fuses with the oblique transverse ligament (OTL) at center eyelid and thereafter both travel to an insertion on the anterior lacrimal crest. Note that maximum vertical eyelid displacement minimally influences the position of the septal ligaments.

The age range of specimens was 71 to 83 years (mean = 75 years), all but 1 of which were male. The anterior cadaver septum consisted of (1) a loose anterior septal membrane (ASM) deep to the postorbicular fascia and (2) 3 discrete ligaments immediately deep to the ASM (Figures 1, 2). The membrane descended from the STL (described below) to the arcus marginalis, mirroring Whitnall's findings (Figures 1, 2). Membrane density ranged from opaque to transparent (Figures 1, 2). Although typically thin inferomedially, regional hernia or dehiscence of the ASM was not observed in any specimen.

The superior ligamentous element, termed the STL (Figure 1), measured 2.0 to 4.0 mm in width and crossed the orbit 4.0 to 6.0 mm below the tarsus. Attaching to the orbital margins contiguous to the lateral and medial canthal tendons, its position was static during passive vertical lid movement (Figure 2). Adipose tissue did not extend above the STL.

**Figure 3.**
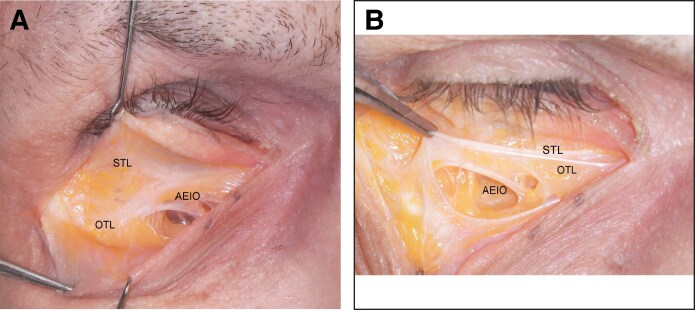
(A) The arcuate expansion of the inferior oblique (AEIO) arises from the medial border of the oblique transverse ligament (OTL) and follows a downward arc to the inferior oblique fascia. The anterior septal membrane, in this specimen demonstrating marked atrophy (seen as vertical light reflections near the arcus marginalis), covers the ligamentous septum and fuses with the superior transverse ligament (STL). (B) Lateral traction on the septal ligaments enhances definition of their medial insertions.

An additional fibrous component, termed the oblique transverse ligament (OTL), arose from the arcus marginalis of the inferior lateral orbital rim in 60% of the cadaver lids and the temporal inferior rim in the remainder. It fused with the STL at mid-eyelid because it crossed the orbit obliquely to the anterior lacrimal crest (Figures 1, 2, 3B). The ligament penetrated deeply into the fat (5.0 mm) in 3 cases but was largely superficial in the remainder. Presumably, the OTL is the arcuate expansion reported in earlier anatomic studies.^14^

A distinct ligamentous branch arose from the medial border of the OTL at roughly mid-eyelid and, after a brief upward arch, descended to the fascial sheath of the inferior oblique muscle (Figure 3A) in the inferomedial orbit. It occurred in all cadavers, and it is likely the AEIO described by Weinberg and Baylis.^16^ Figure 3B demonstrates the medial course of the septal ligaments.

### Operative

The inclusion criteria were patients presenting for lower eyelid aesthetic deformity that subsequently underwent subperiosteal midface lift by the senior author. Patients with global lower lid edema, significant medical conditions, and active smokers were excluded. Ages ranged from 35 to 82 years with a median age of 63 years. The cohort included 58 women, of whom 2 were African American and 6 men.

Four distinct lower eyelid distention patterns were observed in the operative group: total (29/64), clefted (23/64), isolated inferomedial (11/64), and isolated superotemporal (1/64). No more than 2 discrete bulges occurred on any lid. The viscoelastic characteristics of septal constituents identified in the cadavers varied significantly among patients. Specific patterns of fibrous density variance were identified that correlated precisely with the form and location of eyelid surface bulges. As in the cadavers, no septal hernia or dehiscence was observed.

**Figure 4.**
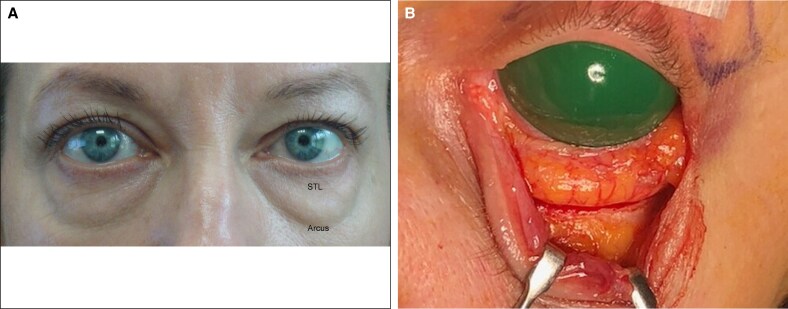
(A) A 54-year-old woman with total lower eyelid distention. The adipose tissue is bounded superiorly by the superior transverse ligament (STL) and inferiorly by the arcus marginalis. The oblique transverse ligament is vestigial. (B) This image demonstrated the appearance of the septum and underlying adipose with complete lower lid distention without clefting.

The most common deformity, total distention, consisted of a single bulge extending from the STL to the arcus marginalis (*n* = 29; Figure 4A). Observed septal characteristics included robust STLs, attenuated OTLs, and translucent ASMs (Figure 4B).

**Figure 5.**
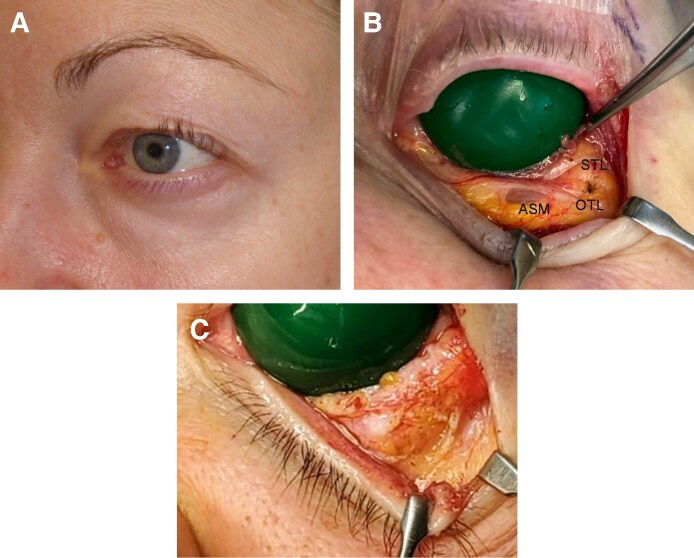
(A) A 75-year-old woman with clefted anterior lamella resulting from advanced atrophy of the entire anterior septal membrane (ASM) with compression of the resulting fat bulge by a firm oblique lateral ligament (OTL). (B) The operative anatomy of the clefted lid demonstrates the diaphanous character of the ASM and firm OTLs and superior transverse ligaments. (C) Division of the OTL effaces adipose tissue compression, allowing the fat to bulge in a pattern like total distention (see Figure 4A).

Clefted distention, marked by 2 discrete bulges separated by an oblique transverse cleft, was next in frequency (*n* = 23; Figure 5A). In contrast to total distention, the superior transverse and oblique lateral ligaments were both robust, and the anterior membrane was atrophic. The OTL projected into the anterior fat 2 to 3 mm, producing the characteristic surface indention (Figure 5B). Division of the OTL effaced the indentation (Figure 5C).

**Figure 6.**
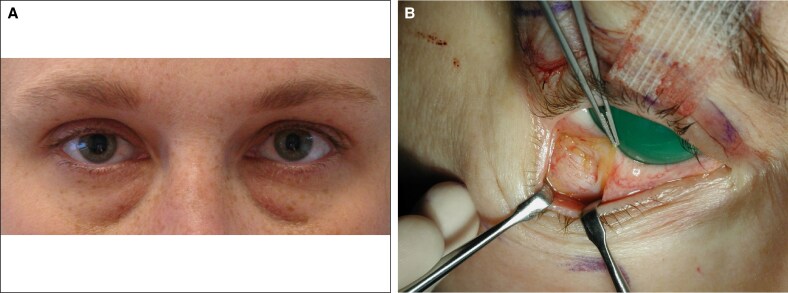
(A) A 37-year-old woman with isolated medial distention, which reflects regional anterior septal membrane (ASM) attenuation. The atrophic medial ASM combined with a firm oblique lateral ligament and thick temporal ASM are associated with this deformity. (B) The temporal ASM is shown to be composed of contiguous horizontal bands.

Isolated medial distention occurred in younger patients (mean age 41 years; *n* = 11; Figure 6A). Operative findings included inferomedial ASM thinning, firm OTLs, and dense lateral ASM fibrosis (Figure 6B).

**Figure 7.**
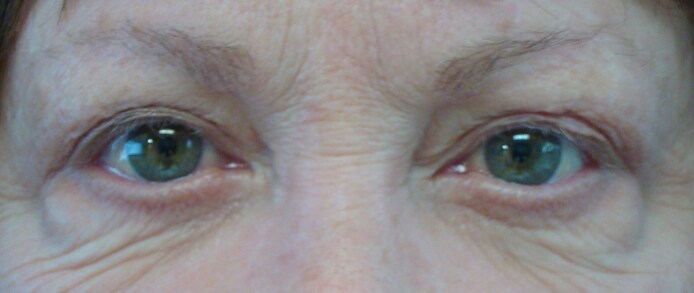
A 73-year-old woman with isolated superolateral distention, another manifestation of regional anterior septal membrane atrophy. The bulge is confined to the space between the origin of the oblique lateral ligament and the lateral superior transverse ligament.

The least common pattern (*n* = 1), isolated superotemporal distention, demonstrated isolated ASM atrophy in the space bounded inferiorly by the origin of the OTL and superiorly lateral STL (Figure 7).

**Figure 8.**
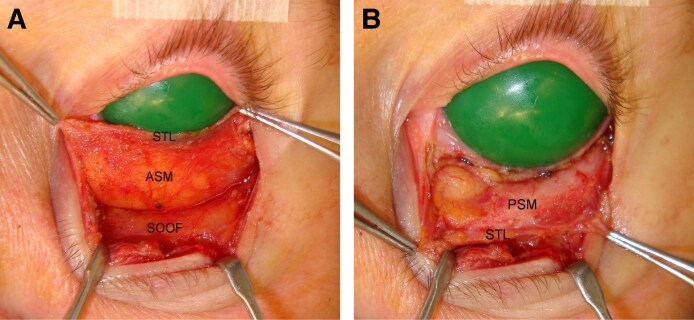
(A) The anterior septal membrane (ASM) descends from the superior transverse ligament (STL) to the arcus marginalis, forming the anterior component of the septal compartment. (B) Release of the STL's osseous attachments gives access to the retro-adipose space, revealing a posterior septal membrane (PSM). Further release of the loose areolar layer separating the PSM from the CPF allows anterior and caudal displacement of the intact septal compartment to well beyond the inferior orbital rim.

Study of the retro-adipose space followed that of the anterior septum in each cohort. Release of the medial and lateral STL attachments to the orbital rims and separation of the fat from the capsulopalpebral fascia (CPF) disclosed a membrane covering the posterior adipose surface. Its density varied among cohorts, being thin medially in all cases (Figure 8A) and laterally in half. The anterior (Figure 8B) and posterior septal membranes fused at the STL, forming a discrete adipose tissue compartment (Figure 8A, B). Continued caudal release of the adipose tissue from the CPF allowed inferior translocation of the septal compartment well beyond the inferior orbital rim (Figure 8B).

**Figure 9.**
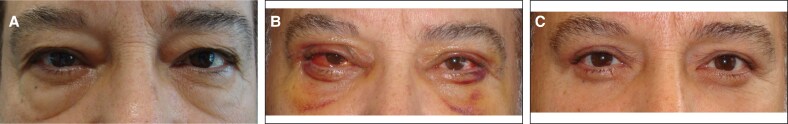
(A) This 73-year-old man has total distention deformity. (B) Six days postoperative from transconjunctival midface lift with subperiosteal fat transposition. (C) Eighteen months postoperative from transconjunctival midface lift with subperiosteal fat transposition.

The septal compartment also contained the origin of the inferior oblique muscle, but connections to other orbital ligamentous structures were not identified. The muscle departed the compartment inferomedially to continue posteriorly near the CPF (Figure 9). Mean postoperative follow-up was 12 months, with chemosis being the only side effect. All cases were resolved with treatment, as depicted in Figure 9A-C.

## DISCUSSION

Eyelid topography is the consequence of the septal constituents' viscoelastic properties because they bear on the underlying tissue. Attenuation of the ASM, whether regional or total, is requisite to eyelid distention. We did not encounter any cases of septal dehiscence or herniation in either cadaveric dissection or operative patients and believe that common patterns of lower lid distention are secondary to the relative viscoelastic properties of septal anatomy. Understanding the relationship between septal anatomy and visualized topography can aid in operative planning.

In the interest of clarity, we chose terms for septal constituents that reflect course, position, or effect. The OTL, for example, in early reports was termed both the arcuate expansion and the superior anterior transverse ligament. For similar reasons, to distinguish it from coexisting anterior lamellar changes, distension seemed an accurate descriptor for eyelid bulging due to fat.

The OTL's tensile strength plays a decisive role in clinical pattern development, because distention occurs medial and/or lateral to a robust OTL and depends on the locus of ASM atrophy (Figures 5A, 6A). OTL atrophy with generalized anterior membrane attenuation results in total eyelid distention (Figure 4A). Ligamentous compression causes a clefted appearance of the adipose tissue but was not associated with actual discrete subcompartments.

Awareness of the underlying anatomy of specific lower eyelid distention patterns allows assessment of the fibrous character of the septum. Clefted distention, for example, implies a diaphanous ASM and robust OTL. Total distension without surface subdivision points to both OTL and ASM atrophy. In both cases, operative procedures relying on septal tensile strength may not achieve long-term correction.

**Figure 10.**
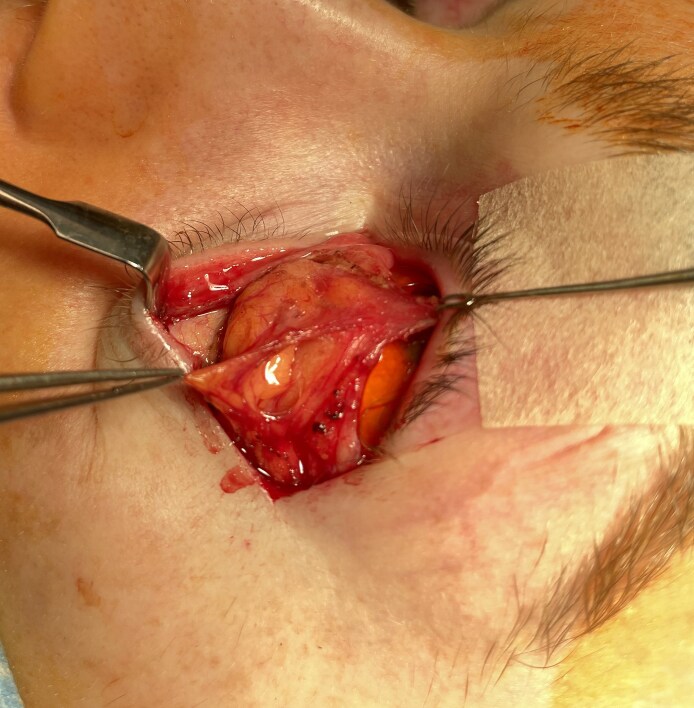
Separating the anterior septal membrane (ASM) from the superior transverse ligament exposes the superior margin of the adipose tissue within the septal compartment of a 58-year-old woman. Continued medial release of the ASM begins exposure of the entire eyelid adipose tissue.

**Figure 11.**
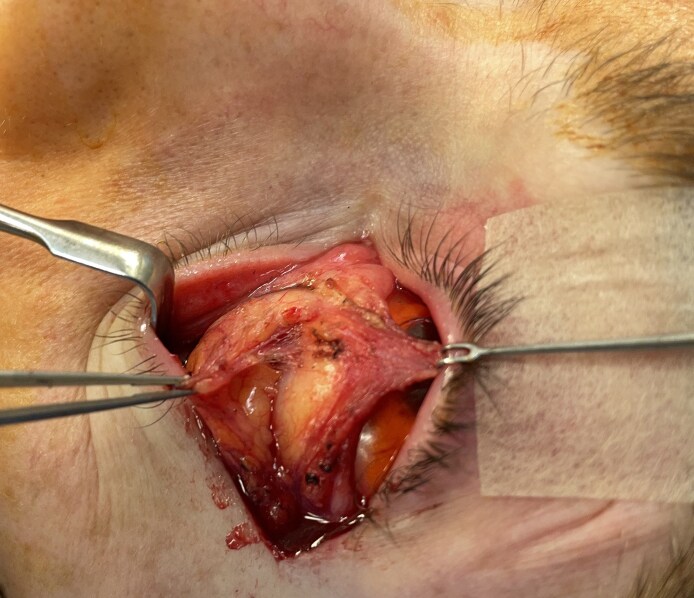
Superior traction on the CPF with countertraction on the anterior septal membrane exposes the loose areolar tissue connecting the fat to the CPF. As the areolar tissue is divided, fat within the septal compartment is displaced toward the inferior orbital rim. The inferior oblique muscle lies just below the areolar tissue.

The architecture of the septal compartment may potentially benefit execution when operative rejuvenation calls for lipectomy or pedicled fat transfer. Opening the ASM just inferior to the STL reveals the superior aspect of the eyelid's adipose tissue (Figure 10). Thereafter, with superior traction applied to the CPF and counter traction to the ASM, division of the loose areolar tissue between the fat and CPF allows cephalad to caudal displacement of the fat to the orbital rim (Figure 11).

**Figure 12.**
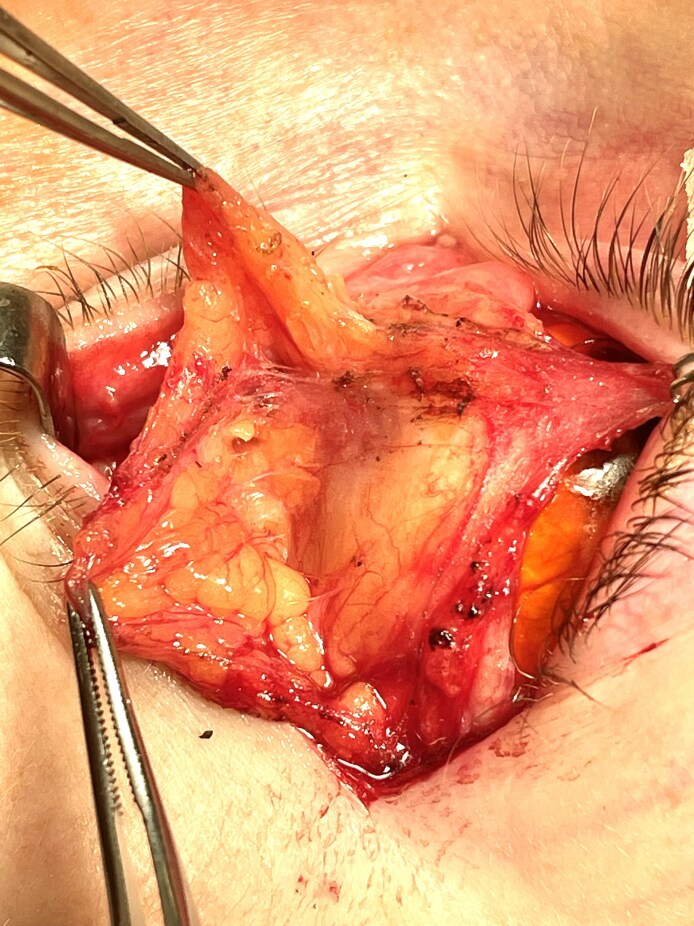
Complete displacement of the septal compartment fully exposes the CPF and the inferior oblique muscles as it penetrates its medial aspect.

**Figure 13.**
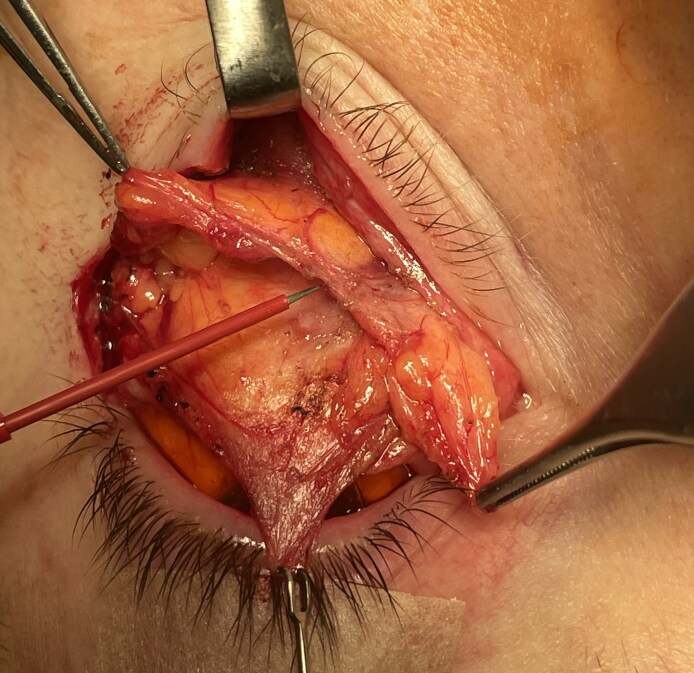
A predictable cleavage plane between the medial and lateral orbital fat permits its separation into 2 discrete units. Adipose tissue separation reveals the CPF and inferior oblique muscle. At this point the fat may be resected or transposed to the subperiosteal space, based on operative goals.

**Figure 14.**
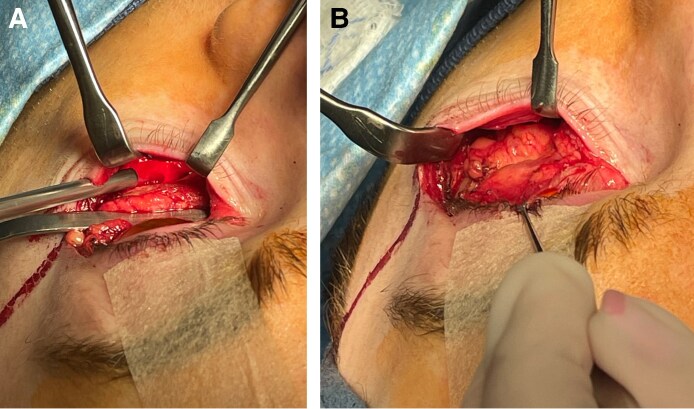
(A) A malleable retractor restrains the orbital fat, revealing the upper maxilla and infraorbital nerve. (B) The entire contents of the septal compartment have been draped over the orbital rim, straddling the nerve. The CPF and inferior oblique lie superior to the adipose tissue.

During these maneuvers, fat remains within the compartment which, unlike piecemeal fat dissection, minimizes bleeding and aids visualization of the inferior oblique muscle (Figure 12). Further, the compartmentalized fat cleaves into 2 discrete structures in a transverse oblique plane mirroring that of the inferior oblique (Figure 13A, B). At the orbital rim, the adipose tissue may be transposed to the premaxillary subperiosteal space or resected, as procedure objectives dictate (Figure 14A, B). This method additionally appeared to minimize vascular trauma to the adipose tissue, perhaps improving long-term pedicle survival.

Features of the septal compartment may also pose potential risk to ocular motility.^21^  ^,22,26^ As can be seen in Figure 3A and B, the OTL connects to the inferior oblique muscle through the AEIO. Traction on either during septal reset and/or fat transfer could limit muscle function. Similarly, the inferior oblique is in close proximity to the CPF, and the latter arises from the inferior rectus muscle. Transposition of a CPF and orbital fat composite, as reported to correct the tear trough deformity, may restrict the action of the oblique or inferior rectus (Figure 9).^24^

Although this study serves to add anatomic detail and discuss clinical implications, it has several limitations. The cadavers utilized were not preserved, which made the anatomic findings more clinically relevant because the dissections more closely mirrored operative anatomy. However, the cadavers were of advanced age and mostly male. In the clinical part of our study, limitations stem largely from a relatively small sample size and selection bias in the form of patients presenting for correction of advanced deformity. The anatomic variation observed is demonstrated in the photographs and was found to relate to preoperative eyelid topography in a consistent manner. Analysis of a larger, more diverse patient population may explain findings reported by other studies and is an area for potential future addition to the literature and our understanding of the anatomy and its clinical implications.

## CONCLUSIONS

This investigation presents the 3D architecture of the inferior orbital septum and considers its potential clinical implications. It refocuses earlier concepts regarding the surface appearances of aging lower eyelids and underlying anatomic etiology. Although we discuss operative technique, potential complications, and results, we believe that technique and patient selection must be individualized to each patient and may be surgeon specific. There is still room for further study of lower lid anatomy to facilitate further advances in techniques and outcomes for correcting the appearance of the aged lower lid.
